# Dentifrices for children differentially affect cell viability in vitro

**DOI:** 10.1007/s00784-016-1813-4

**Published:** 2016-04-06

**Authors:** Barbara Cvikl, Adrian Lussi, Andreas Moritz, Reinhard Gruber

**Affiliations:** 1 Department of Preventive, Restorative and Pediatric Dentistry, School of Dental Medicine, University of Bern, Freiburgstrasse 7, CH-3010 Bern, Switzerland; 2 Department of Conservative Dentistry & Periodontology, Medical University of Vienna, Sensengasse 2a, A-1090 Vienna, Austria; 3 Department of Oral Biology, Medical University of Vienna, Sensengasse 2a, A-1090 Vienna, Austria

**Keywords:** Children, Toothpaste, Dentifrices, Cytotoxicity, In vitro

## Abstract

**Objectives:**

Child dentifrices vary in their composition, with possible differential impacts on cells in the oral soft tissue. While cytotoxicity studies have been performed on adult dentifrices, no respective studies have thus far been reported on child dentifrices.

**Material and methods:**

Seventeen commercial dentifrices for children up to 12 years of age were evaluated with respect to their in vitro cytotoxicity on gingival fibroblasts, oral squamous cell carcinoma HSC-2 cells, and L929 mouse fibroblasts. Proliferation was analyzed and live-dead staining was performed.

**Results:**

Ten child dentifrices greatly reduced cell viability with LC50 values below 5 %. Four dentifrices showed a moderate cytotoxicity with LC50 values between 5 and 20 %. Three child dentifrices showed almost no cytotoxicity with LC50 values above 95 %. The results of the assays for proliferation and live-dead staining supported these findings.

**Conclusions:**

The different composition of the child dentifrices translated into a broad spectrum of in vitro cytotoxicity on cells of the oral cavity.

**Clinical relevance:**

The in vitro data provide the scientific foundation for further in vivo research testing the clinical relevance of the present findings.

## Introduction

Children with caries in their primary teeth suffer three times more from caries in their permanent teeth than their peers of the same age [1]. The most important factor in preventing the development of caries and reducing its incidence is regular and supervised tooth brushing with fluoride dentifrices [2]. Children, however, are neither motivated nor skilled enough to brush their teeth effectively [3]. Consequently, parents ideally supervise their children’s tooth brushing or brush their teeth when they are very young. This is also important since young children ingest up to 65 % of the toothpaste [4, 5] due to a not-yet mature swallowing reflex [6]. Despite the recommendations to use a pea-sized amount of toothpaste for one tooth-brushing event, parents may use a larger amount [3]. The need for special child dentifrices is further supported by the fact that a majority of mothers use the same toothpaste for their children as for themselves [5]. Furthermore, it is shown that brushing time is prolonged when the dentifrices is good tasting and a pleasant consistency [7]. Changing behavioral factors at a young age toward good hygienic practices has been shown to lead to better oral health for a lifetime [8]. But the taste and the consistency of toothpaste are not the only differences between available products for adults and children. The ideal product for children should provide maximal fluoride availability, minimal abrasivity, and ingredients that will not interfere with fluoride delivery and assure a pleasant brushing experience [9]. It should also be distinguished if the children already have a mixed dentition. With a mixed dentition toothpastes should perform higher levels of foam, higher amounts of fluoride and other tastes [9]. However, the effect of dentifrices and their ingredients on cells of the oral cavity may also be important, since they are in direct contact with these respective tissues during and also after brushing.

To avoid the compromising of child’s health, the American and European Academies of Pediatric Dentistry (AAPD and EAPD) published recommendations for the correct use of dentifrices [10]. The recommendations refer only to the fluoride content, since the beneficial and also potential adverse effects of fluorides have been well investigated [2]. The main fluoride intake of young children occurs through ingestion of fluoridated dentifrices [11, 12]. In order to reduce the risk of fluorosis in developing permanent teeth, international recommendations are the use of 1000 ppm of fluoride for children under 6 years and up to 1500 ppm for older children [13]. Fluoride, however, is not the only ingredient that is ingested by children when toothpaste is swallowed. Dentifrices contain a wide range of different ingredients, each with a special purpose, but some also with the potential to negatively affect the oral mucosa.

The oral mucosal sensitivity and contact stomatitis are reported to arise as a result of different toothpaste ingredients such as abrasives, detergents, binding agents, humectants, preservatives, coloring agents, antiseptics, fluoride salts, and flavorings in sensitive individuals [14]. In particular, triclosan, known for its antibacterial and antiplaque effect, and sodium lauryl sulfate, a common detergent, are cited as offending ingredients. Clinical intraoral adverse effects such as burning mouth sensations, epithelial desquamation, and recurrent aphtous ulcerations [15, 16], as well as reduced cell viability in in vitro investigations point to possible issues with the diverse ingredients used in adult dentifrices [17]. No study so far has examined the influence of child dentifrices on cells of the oral cavity. Therefore, the aim of this study was to investigate the effect of 17 commercial child dentifrices on cells of the oral mucosa and on cells commonly used for cytotoxicity testing. The working hypothesis was that the soluble compounds of dentifrices especially designed for children do not change cell viability compared to the control.

## Material and Methods

### Cell culture and stimulation of cells

Epithelial cells and gingival fibroblasts, which may come in contact with toothpaste in the oral cavity, as well as a L929 cell line which is commonly used for cytotoxicity testing, were used for the main experiments on cell viability. Furthermore, gingival fibroblasts were used in indicated experiments for live-dead cell staining and proliferation assays. For experiments with primary cells (gingival fibroblasts), cell pools made from cells of three different donors were used to minimize donor variability. Cells were prepared from tissue grafts after wisdom tooth extraction in healthy individuals. Before the extraction, the patients were informed about the possibility of using their teeth together with adhering tissue for research purposes and consent was obtained (Kantonale Ethikkommission Bern). Oral squamous cell carcinoma cell line HSC-2 (the source of epithelial cells) was kindly provided by Dr. Rausch-Fan from the Medical University of Vienna, Austria. Murine L929 fibrosarcoma cells were kindly provided by Dr. Erik Hedbom, School of Medical Dentistry, University of Bern, Switzerland. Cells were cultivated in a humidified atmosphere in Dulbecco’s Modified Eagle Medium (DMEM, Invitrogen Corporation, Carlsbad, CA, USA) supplemented with 10 % fetal bovine serum (FCS; PAA Laboratories, Linz, Austria) and antibiotics (Invitrogen) at 37 °C and 5 % CO_2_. For experiments on cell viability and proliferation assays, cells were seeded in microtiter plates (Greiner Bio-One GmbH, Frickenhausen, Germany) at 30,000 cells/cm^2^ 1 day before stimulation with the soluble compounds of child dentifrices. For live-dead cell staining, cells were seeded onto chamber slides (Thermo Scientific Nunc, Waltham, MA, USA) under the same conditions as the other assays.

### Stimulation with the soluble compounds of child dentifrices

The child dentifrices investigated in the study were Blendi up to 6 years (Blend-a-Med, Procter & Gamble Co., Cincinnati, OH, USA), Candida Kids up to 6 years (Mibelle AG, Switzerland), Candida Junior 6–12 years (Mibelle AG, Switzerland), Colgate® 2–6 years (Colgate-Palmolive, New York, NY, USA), Dontodent Kids up to 6 years (dm-drogerie markt GmbH, Karlsruhe, Germany), Dontodent Junior 6 years plus (dm-drogerie markt GmbH, Karlsruhe, Germany), Elmex® up to 6 years (GABA International AG, Colgate-Palmolive), Elmex® Junior 6–12 years (GABA International AG, Colgate-Palmolive), Mentadent Kids up to 6 years (Unilever Austria GmbH, Vienna, Austria), nenedent®baby 0.5–2 years (Dentinox, Berlin, Germany), nenedent® Kinderzahncreme up to 6 years (Dentinox), Odol-med3® Milchzahn 0.5–5 years (GlaxoSmithKline, Brentford, UK), Odol-med3® Milchzahn up to 6 years (GlaxoSmithKline), Odol-med3® Junior 6 years plus (GlaxoSmithKline), Sensodyne® Junior 6 years plus (GlaxoSmithKline), mein kleines Theramed up to 6 years (Henkel AG & Co. KGaA, Düsseldorf, Germany), and Theramed Junior 6 years plus (Henkel AG & Co. KGaA). Detailed information on the dentifrices used in the study is given in Table 1. In order to extract the soluble compounds of the toothpaste, toothpaste slurries were made with serum-free medium (50 % *w*/*v*) in sealable plastic tubes with a magnetic stirrer bar at 350 rpm. Afterwards, the slurry was centrifuged at 16,000×*g* for 10 min and soluble compounds (toothpaste-conditioned medium; TCM) were collected and filter sterilized as described elsewhere [17]. Sensodyne® Junior 6, Theramed −6, and Blendi −6 were diluted less (80 % *w*/*v*), since the calculation of the half lethal concentration (LC50) was not possible otherwise. Directly before stimulation of the cells, 50 and 80 % toothpaste-conditioned medium was further diluted up to a final concentration of 0.4 %.

**Table 1:** Dentifrices used in the present study

Toothpaste (Company)	Detergents	Composition	Expiry date	Country of purchase
Blendi blend-a-med −6 (Procter & Gamble UK, Weybridge, KT13 OXP, UK)	Cocamidopropyl Betaine	Aqua, Aroma, Benzyl Alcohol, Carbomer, Cochineal Red, Hydrated Silica, Mica, Sodium Chloride, Sodium Saccharin, Sodium Fluoride (500 ppm), Sodium Phosphate, Sorbitol, Titandioxid, Trisodium Phosphate, Xanthan Gum	01/2016	Austria
Candida Kids 0–6 (Migros, Mibelle AG, Buchs, Switzerland)	Sodium-Coco-Sulfate	Aqua, Aroma, Calcium Glycerophosphate, Cellulose Gum, Citral, Hydrated Silica, Hydrogenated Starch Hydrolysate, Iron Oxides Limonene, Linalool, Mica, Sodium Hydroxide, Sodium Saccharin, Sodium Monofluorphosphate (500 ppm), Titandioxid	09/2015	Switzerland
Candida Junior 6–12 (Migros, Mibelle AG, Buchs, Switzerland)	Sodium-Coco-Sulfate	Aqua, Aroma, Cellulose Gum, Dicalcium Phosphate, Hydrated Silica, Hydrogenated Starch Hydrolysate, Limonene, Sodium Hydroxide, Sodium Saccharin, Sodium Monofluorphosphate (1400 ppm), Titandioxid	01/2016	Switzerland
Colgate® 2–6 (Colgate-Palmolive, New York, NY, USA)	Sodium Lauryl Sulfate	Aqua, Aroma, Brilliant Blue FCF, Cellulose Gum, Glycerin, Hydrated Silica, Limonene, Mica, Polyethylene, Polyethylenglycol, Sodium Fluoride (1000 ppm), Sodium Saccharin, Sorbitol, Titandioxid	08/2015	Austria
Dontodent^DM^ Kids −6 (dm-drogerie markt, Karlsruhe, Germany)	Cocamidopropyl Betaine, Sodium C14–16 Olefin Sulfonate	Aqua, Aroma, Cellulose Gum, Cochineal Red Hydrated Silica, Mica, Sodium Chloride, Sodium Saccharin, Sodium Fluoride (500 ppm), Sorbitol, Titandioxid	09/2016	Austria
Dontodent^DM^ Junior 6 plus (dm-drogerie markt, Karlsruhe, Germany)	Sodium C14–16 Olefin Sulfonate	Aqua, Aroma, Brilliant Blue FCF, Calcium Glycerophosphate, Hydrated Silica, Limonene, Mica, Sodium Saccharin, Sodium Fluoride (1000 ppm), Sorbitol, Titandioxid, Xanthan Gum	09/2016	Austria
Elmex®Kinderzahnpasta −6 (GABA International AG, Colgate-Palmolive)	Cocamidopropyl Betaine, Aminfluorid (500 ppm)	Aqua, Aroma, Hydrated Silica, Hydrochoric Acid, Hydroxyethylcellulose, Limonene, Sodium Saccharin, Sorbitol, Titandioxid	12/2015	Austria
Elmex® Junior 6–12 (GABA International AG, Colgate-Palmolive)	Aminfluorid (1400 ppm)	Aqua, Aroma, Hydrated Silica, Hydrochoric Acid, Hydroxyethylcellulose, Limonene, Sodium Saccharin, Sorbitol/Glycerin, Titandioxid	12/2015	Austria
Mentadent Kids 3–6 (Unilever Dept ER Wirral, JW, UK)	Sodium Lauryl Sulfate	Aqua, Aroma, Calcium Gluconate, Cellulose Gum, Glycerin, Hydrated Silica, Phthalocyanine Blue, Polyethylenglycol, Sodium Saccharin, Sodium Fluorid (1000 ppm), Sorbitol, Titandioxid, Tocopheryl Acetate	11/2015	Austria
nenedent®baby 0.5–2 (Dentinox®, Berlin, Germany)	Sodium Lauryl Sarcosinate	Aqua, Aroma, Disodium EDTA, Glycerin, Hydrated Silica, Propylene Glycol, Sodium Monofluorphosphate (500 ppm), Sodium Chloride, Titandioxid, Xanthan Gum, Xylitol	08/2015	Austria
Nenedent® -6 (Dentinox®, Berlin, Germany)	Sodium Lauryl Sarcosinate	Aqua, Aroma, Disodium EDTA, Glycerin, Hydrated Silica, Propylene Glycol, Sodium Monofluorphosphate (500 ppm), Sodium Chloride, Titandioxid Xanthan Gum, Xylitol	08/2016	Austria
Odol-med3® 0.5–5 (GlaxoSmithKline, Brentford, UK)	Cocamidopropyl Betaine, Sodium Methyl Cocoyl Taurate	Aqua, Aroma, Carrageenan, Glycerin, Hydrated Silica, Limonene, Phthalocyanine Blue, Sodium Saccharin, Sodium Fluorid (500 ppm), Sorbitol, Thioindigo, Titandioxid, Xanthan Gum	01/2016	Austria
Odol-med3® 0–6 (GlaxoSmithKline, Brentford, UK)	Sodium Methyl Cocoyl Taurate	Aqua, Aroma, Disodium Phosphate, Glycerin, Hydrated Silica, Phthalocyanine Blue, Methylparaben, Polyethylenglycol, Propylparaben, Sodium Saccharin, Sodium Fluorid (500 ppm), Thioindigo, Titandioxid, Xylitol, Xanthan Gum	01/2016	Austria
Odol-med3® Junior 6 plus (GlaxoSmithKline, Brentford, UK)	Cocamidopropyl Betaine, Sodium Methyl Cocoyl Taurate	Aqua, Aroma, Carrageenan, Glycerin, Hydrated Silica, Limonene, Phthalocyanine Blue, Sodium Saccharin, Sodium Fluorid (1450 ppm), Sorbitol, Thioindigo, Titandioxid, Xanthan Gum	10/2015	Austria
Sensodyne® Junior 6 plus (GlaxoSmithKline)	Cocamidopropyl Betaine	Aqua, Aroma, Glycerin, Hydrated Silica, Limonene, Polyethylenglycol, Sodium Fluorid (1450 ppm), Sodium Hydroxide, Sodium Saccharin, Sorbitol, Sucralose, Titandioxid, Xanthan Gum	08/2015	Austria
Theramed Junior 1–6 (Henkel AG, Düsseldorf, Germany)	Cocamidopropyl Betaine	Aqua, Aroma, Azorubine, Calcium Glycerophosphate, Cellulose Gum, Disodium Phosphate, Glycerin, Hydrated Silica, Methylparaben, Sodium Chloride, Sodium Fluorid (500 ppm), Sodium Saccharin, Sorbitol	09/2015	Austria
Theramed Junior 6 plus (Henkel AG, Düsseldorf, Germany)	Sodium Lauryl Sulfate, Cocamidopropyl Betaine	Aqua, Aroma, Azorubine, Calcium Glycerophosphate, Disodium Phosphate, Glycerin, Hydrated Silica, Propylen Glycol, Mica, Methylparaben, Sodium Chloride, Sodium Fluorid (1000 ppm), Sodium Saccharin, Sodium Sulfate, Sorbitol, Titandioxid, Xanthan Gum	09/2015	Austria

### Cell viability and calculation of the half lethal concentration

The TCM of 17 different child dentifrices was used at concentrations of 50 and 80 % and diluted up to a final concentration of 0.4 % for stimulating gingival fibroblasts (GF), epithelial cells (HSC-2), and murine fibrosarcoma cells (L929). After 2 min of stimulation, the recommended as well as applied average time for tooth brushing [18, 19], cells were washed with phosphate-buffered saline (PBS) to neutralize the further effects of the TCM on the cells, and serum-free media was added containing MTT (3-[4,5-dimethythiazol-2-yl]-2,5-diphenyltetrazolium bromide, 0.5 mg/ml, Sigma-Aldrich, St. Louis, MO, USA) for 2 h at 37 °C. Optical density of formazan crystals, formed by NAD(P)H-dependent oxidoreductases and dissolved in dimethyl sulfoxide, was measured with a microplate reader (EL 808, Biotek Instruments, Winooski, VT, USA) and normalized to untreated cells. The LC50 was calculated by an exponential regression analysis using the formula y = m*e^(b*x) as described elsewhere [17]. Furthermore, toothpaste slurries (25, 12, and 6 % *w*/*v*) were directly used for stimulating oral fibroblasts and subsequent viability measurements were performed in indicated experiments.

### Cell proliferation assay

Cell proliferation was measured by incorporation of 5-bromo-2′-deoxyuridine (BrdU) using the Cell Proliferation ELISA, BrdU (colorimetric) kit from Roche (Basel, Switzerland). The TCM of 17 different child dentifrices, at a concentration of 5 %, was used for stimulating gingival fibroblasts. After 2 min of stimulation, cells were washed with PBS, and serum-free media was added containing 5-bromo-2′-deoxyuridine. After 2 h, the BrdU incorporation was determined according the manufacturer’s instructions and normalized to untreated cells.

### Live-dead cell staining

The TCM of 17 different child dentifrices at a concentration of 5 % was used for stimulating gingival fibroblasts. After 2 min of stimulation, cells were washed with PBS, and a cell permeable green fluorescent dye to stain live cells was added. Dead cells were stained by propidium iodide, a red fluorescent dye, which in viable cells is actively pumped out of the cytoplasm. Visualization was performed using fluorescence microscopy.

### Statistical analysis

Data on the LC50 of the different child dentifrices were reported by the mean and standard deviation of four independent experiments, each performed in duplicate. Differences in LC50 between cells treated with the 17 different TCM were tested using one-way ANOVA followed by a post hoc Tukey-HSD test (SPSS version 19.0, SPSS Inc., Chicago, IL, USA). An alpha of 5 % was considered significant. Data on the cell proliferation (BrdU incorporation) were described by the mean and standard deviation.

## Results

### Child dentifrices differentially affect cell viability

**Fig. 1:**
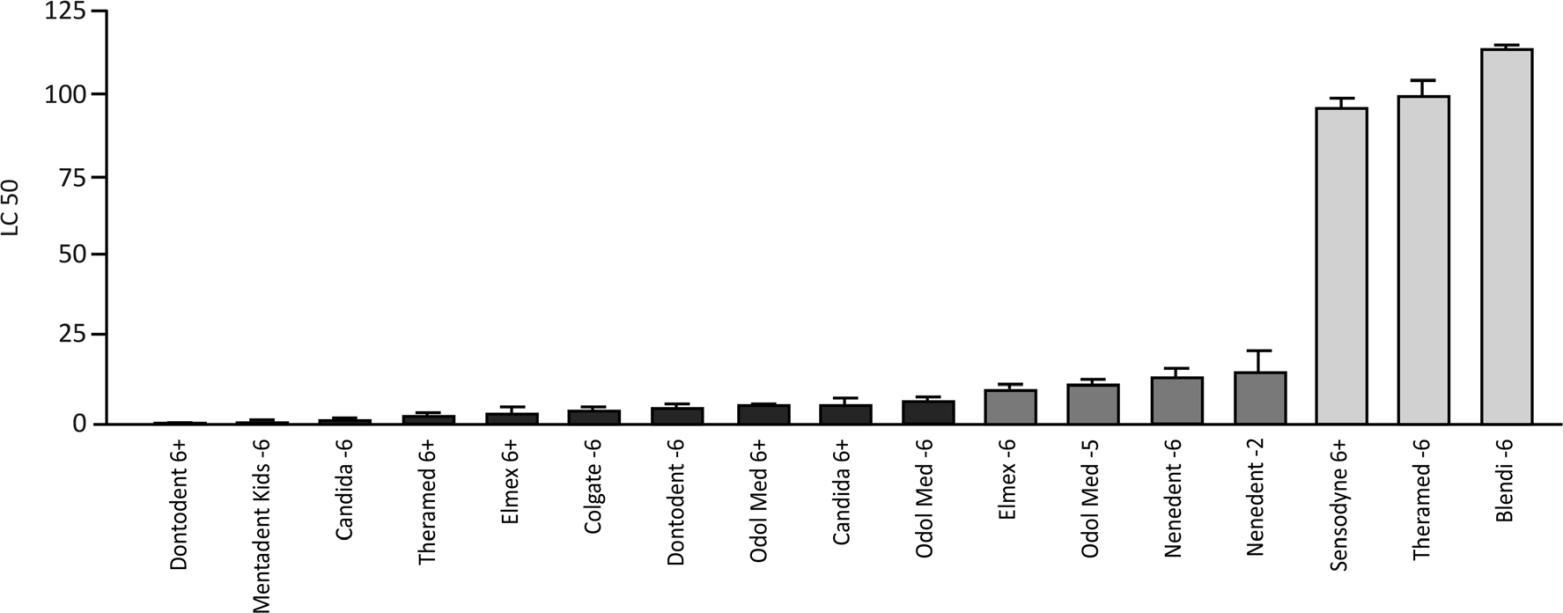
LC50 of gingival fibroblasts after stimulation with different concentrations of the soluble compounds from child dentifrices. Oral fibroblasts were exposed for 2 min to various concentrations of the soluble compounds from child dentifrices. Viability was measured with an MTT assay and the LC50 were calculated, resulting in three groups that differed statistically significantly. Child dentifrices exhibiting an LC50 below 5 % were Dontodent 6+, Mentadent Kids −6, Candida −6, Theramed 6+, Elmex 6+, Colgate −6, Dontodent −6, Odol med 6+, Candida 6+, and Odol med −6 (black bars); child dentifrices exhibiting an LC50 between 5 and 20 % were Elmex −6, Odol med −5, nenedent®baby −2, and Nenedent −6 (dark-gray bars); child dentifrices exhibiting an LC50 above 95 % were Sensodyne 6+, Theramed −6, and Blendi −6 (light-gray bars)

The LC50 data of TCM from 17 different child dentifrices after stimulating gingival fibroblasts for 2 min, measured by the capacity of the cells to convert MTT into formazan crystals, are shown in Fig. 1. Based on these data, three groups of LC50 values showed statistically significant differences: (i) child dentifrices exhibiting an LC50 below 5 % were Dontodent 6+, Mentadent Kids −6, Candida −6, Theramed 6+, Elmex® 6+, Colgate® −6, Dontodent −6, Odol-med3® 6+, Candida 6+, and Odol-med3® −6; (ii) child dentifrices exhibiting an LC50 between 5 and 20 % were Elmex® −6, Odol-med3® −5, nenedent®baby −2, and nenedent® −6; (iii) child dentifrices exhibiting an LC50 above 95 % were Sensodyne® 6+, Theramed −6, and Blendi −6. LC50 data after stimulating epithelial cells (HSC-2) and murine fibrosarcoma cells (L929) for 2 min are shown in Table 2 and confirm the data obtained on gingival fibroblasts. Together, these observations suggest that the majority of child dentifrices substantially reduce the viability of cells. Some child dentifrices, however, affect the viability less or not at all. By comparing dentifrices from the same manufacturers with regard to the age groups for which they are prepared, a consistent picture appeared. Child dentifrices for younger children below 6 years of age resulted in higher LC50 values than their comparable dentifrices for children above 6 years. This is true for Dontodent, Elmex®, Theramed, and Odol-med3®; but not so for Candida.

**Table 2:** LC50 of epithelial cells and murine fibroblasts after stimulation with different concentrations of TCM

	Human Sarcoma Cells		L929	
	Mean	Standard deviation	Mean	Standard deviation
Dontodent^DM^Junior 6+	0.27	0.36	0.57	0.82
Mentadent Kids 3–6	2.04	0.95	2.62	0.67
Candida Kids 0–6	1.07	0.45	1.70	1.12
Theramed Junior 6+	1.52	0.82	2.09	0.51
Elmex® Junior 6–12	6.00	1.40	0.81	0.51
Colgate® 2–6	3.09	0.94	3.52	0.80
Dontodent^DM^ Kids −6	2.44	0.53	2.25	0.45
Odol-med3® Junior 6+	2.53	0.40	2.72	0.97
Candida Junior 6–12	1.32	0.39	2.16	0.96
Odol-med3® 0–6	6.08	1.08	5.12	3.04
Elmex® -6	7.03	4.57	8.70	5.57
Odol-med3® 0.5–5	7.91	0.97	7.04	1.62
Nenedent® -6	9.11	2.29	8.76	1.83
nenedent®baby 0.5–2	7.58	5.22	14.22	10.01
Sensodyne® Junior 6+	110.92	27.13	88.73	7.56
Theramed Junior 1–6	97.26	19.05	97.82	22.89
Blendi blend-a-med −6	107.22	8.74	92.12	8.90

**Fig. 2:**
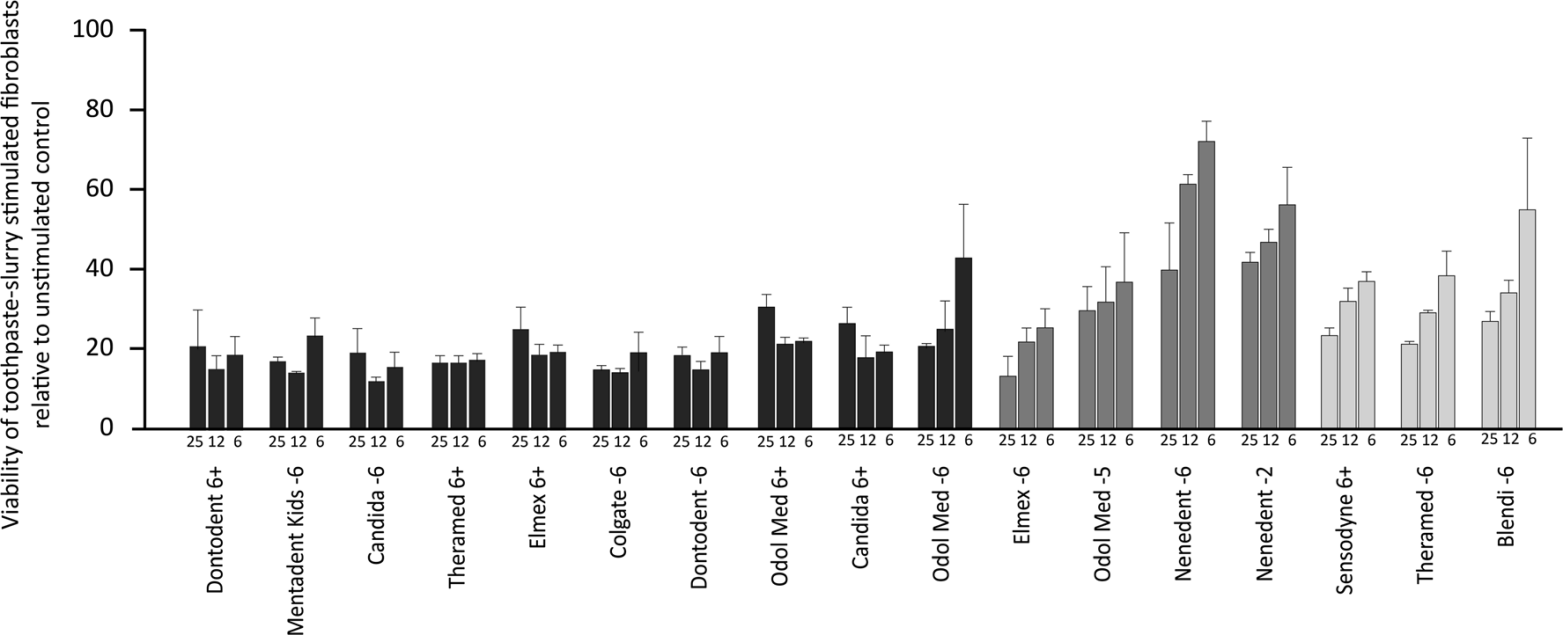
Viability of oral fibroblasts after stimulation with different concentrations of toothpaste slurries from child dentifrices. Oral fibroblasts were exposed for 2 min to 25, 12, and 6 % concentrations of uncentrifuged and unfiltered toothpaste slurries from child dentifrices. Viability was measured with an MTT assay. The 25 and 12 % concentrations of uncentrifuged and unfiltered toothpaste slurries strongly reduced cell viability, almost independent of the toothpaste used. When using a 6 % concentration, the results mainly support the data of the experiments when toothpaste-conditioned medium was used

The results of the viability testing when fibroblasts were stimulated with the uncentrifuged and unfiltered toothpaste slurry are shown in Fig. 2. The 25 and 12 % concentrations of uncentrifuged and unfiltered toothpaste slurries strongly reduced cell viability, almost independent of the toothpaste used. When using a 6 % concentration, the results mainly support the data of the experiments when toothpaste-conditioned medium was used.

### Child dentifrices differentially affect cell proliferation

**Fig. 3:**
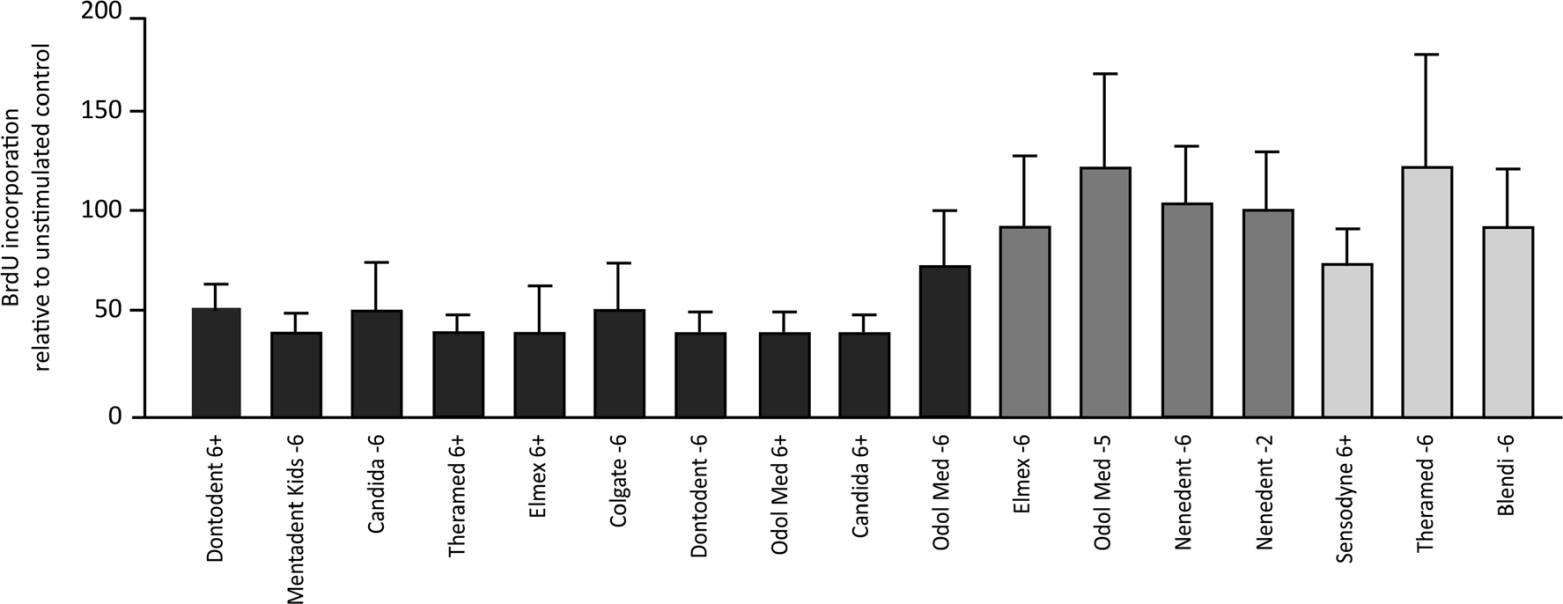
BrdU incorporation by gingival fibroblasts after stimulation with a 5 % concentration of the soluble compounds from child dentifrices. Proliferation was expressed as BrdU incorporation during DNA synthesis. The percentage of BrdU incorporation was normalized to untreated cells. Dentifrices that resulted in LC50 values between 5 and 20 % (*dark-gray bars*) showed in the BrdU incorporation assay similar values to dentifrices that achieved LC50 values of more than 90 % (*light-gray bars*). Dentifrices with LC50 values below 5 % exhibited decreased BrdU values (*black bars*)

In parallel, the incorporation of BrdU in the DNA of gingival fibroblasts after stimulating the cells with 5 % TCM from the 17 different child dentifrices was investigated (Fig. 3). The mean values for the dentifrices that showed an LC50 below 5 % (i) were between 0.4 and 0.7, compared to the unstimulated control with 1.0. Mean values for dentifrices from the group with an LC50 between 5 and 20 % (ii) and above 95 % (iii) were between 0.9 and 1.2 and between 0.7 and 1.2, respectively. Together these data support the results from the viability testing.

### Live-dead cell staining of fibroblasts after stimulation with child dentifrices

**Fig. 4:**
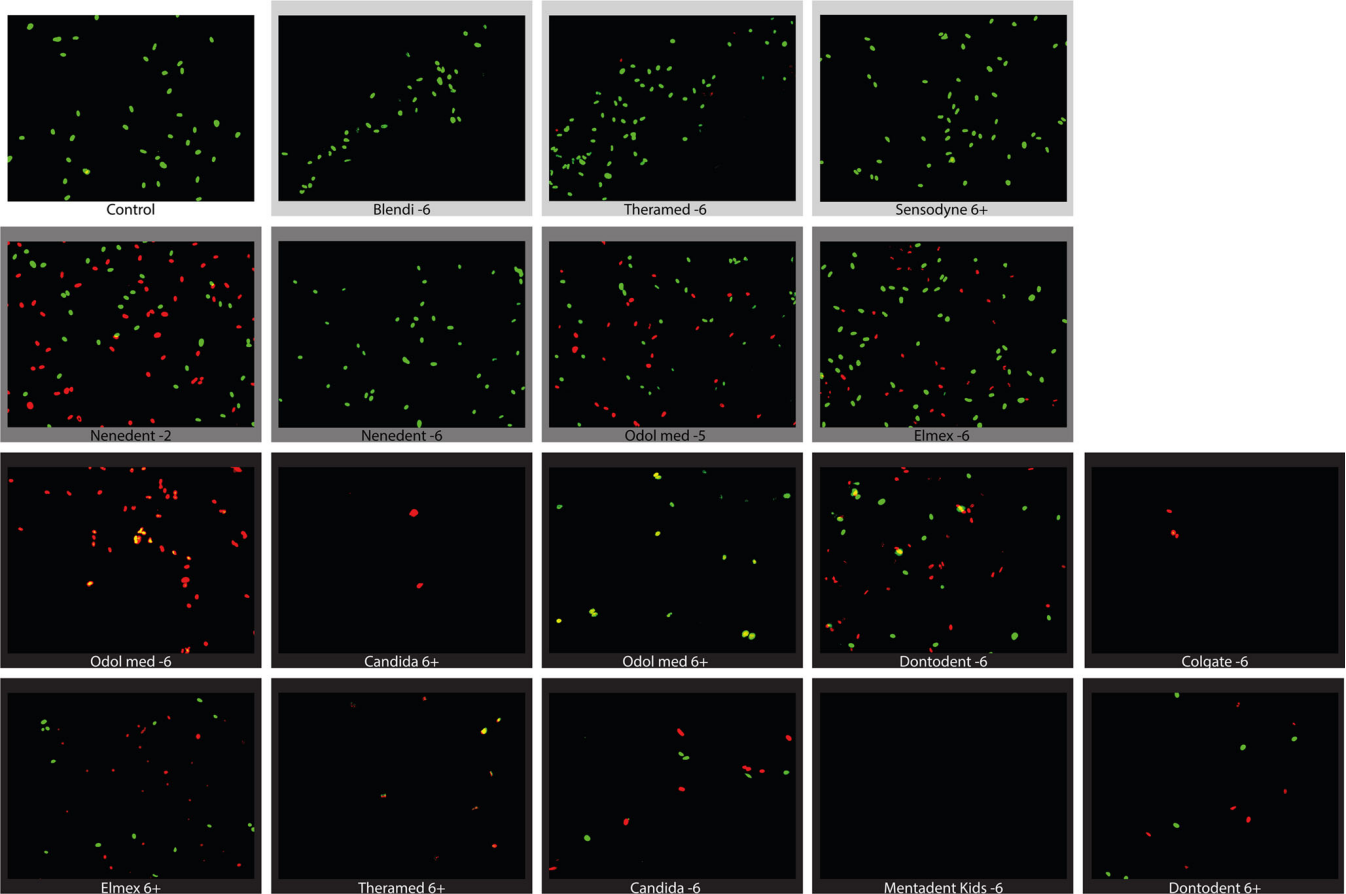
Live-dead cell staining of gingival fibroblasts after stimulation with a 5 % concentration of the soluble compounds from child dentifrices. Oral fibroblasts were exposed for 2 min to a 5 % concentration of the soluble compounds from child dentifrices. Viable cells stained green, dead cells stained red

Consistent with the results of the viability testing and the proliferation assay, stimulation with the TCM of child dentifrices at a concentration of 5 % resulted in different ratios of living cells (green) to dead cells (red) depending on the respective TCM (Fig. 4). Cells stimulated with a TCM from dentifrices with an LC50 above 95 % mostly appeared green, which was similar to the unstimulated control group. In addition, the dentifrices from the groups with an LC50 between 5 and 20 % and an LC50 below 5 % supported the results of the viability and the proliferation assays in the live-dead staining. In the group with an LC50 between 5 and 20 %, green cells were visible beside the red cells. In the group with an LC50 below 5 %, mostly red cells were visible, if any.

## Discussion

The usage of fluoridated dentifrices for daily oral hygiene is a sine qua non for maintaining healthy primary teeth or permanent teeth. The most important factor in preventing the development of caries and reducing its incidence is regular and supervised tooth brushing with fluoride dentifrices [2]. With respect to the particular needs of children and their primary teeth, special dentifrices have been developed [20]. The main differences between child dentifrices and dentifrices for adults are the level and type of surfactants, the type of thickening gums, flavor, color, and the amount of fluoride. While the use of fluoride in dentifrices for children is very well investigated and recommendations by international academies are available [2, 10], no investigations on child dentifrices with respect to cells of the oral cavity have been reported. This is of particular interest since dentifrices for adults have shown significant differences in the LC50 when exposed to gingival fibroblasts [17]. Six out of nine dentifrices exhibited an LC50 below 5 % [17]. These results are in agreement with the present study, which showed that 10 out of 17 child dentifrices exhibited an LC50 below 5 %. However, conclusions on which components mediate the cytotoxic effects cannot be drawn based on the present in vitro setting.

In the present study, sodium lauryl sulfate (SLS)-containing dentifrices exhibited LC50 values below 5 %, which is in agreement with data from adult dentifrices [17]. These results also confirm in vitro experiments on the viability of human keratinocytes after stimulation with SLS [21]. Most child dentifrices, however, contain other detergents. Cocamidopropyl betaine (CAPB), for example, is another detergent often used in child dentifrices. In the present study, CAPB is the single detergent in three child dentifrices that achieved the highest LC50 values, but once it is mixed with another detergent, the LC50 reduced significantly. This finding is consistent with the study on adult dentifrices [17], but contrary to studies showing that CAPB and SLS have a similar cytotoxic effect in vitro [22, 23]. One explanation for this inconsistency could be that unknown and most likely varying concentrations of CAPB were used in the indicated studies. The exact concentrations of detergents in the dentifrices that are harmful for cells will remain unclear until the exact concentrations are reported in future studies.

It is notable that only a few manufacturers use SLS in toothpaste for children compared to adult dentifrices. It is also striking that dentifrices for children up to 6 years show generally higher LC50 values than dentifrices for children over 6 years, even when they are from the same manufacturer (e.g., Dontodent, Elmex®, Theramed, and Odol-med3®, but not Candida). One explanation could be that the concentration of detergents is lower in dentifrices for children up to 6 years, since children dislike foam [9]. Children over 6 years of age with a mixed dentition are moved into adult-type dentifrices with other tastes, a higher amount of fluoride, and also higher levels of foam, which probably means a higher concentration of surfactants [9]. This might be an explanation for the different results on cell viability after stimulation with dentifrices from the same manufacturer but for different age limits.

A limitation of the study is the in vitro design and therefore the results must not be clinically over interpreted. It can be assumed that higher concentrations for the same cytotoxic effects would be necessary in vivo, since neither the salivary flow nor the salivary pellicle was simulated. Furthermore, possible cell protective effects like tissue barriers or immunological aspects have not been considered. Another issue, perhaps with lesser implications, was that the child dentifrices were tested on oral fibroblasts obtained from gingival tissue from adults, instead of from children. We cannot rule out that the metabolic situation in cells from children might be different than the situation in adult’s cells. However, this is not likely to have strongly influenced the results, especially since two other cell lines confirmed the results obtained with fibroblasts. One open issue is that the exact concentrations of detergents in the dentifrices are unknown and it is not possible to verify whether the detergents cause the cytotoxic effect. Furthermore, in the clinical situation, the dentifrices as a whole are in contact with the oral mucosa and not only the soluble ingredients. It might be that further ingredients of the dentifrices, both in the soluble and in the insoluble part, cause cytotoxic effects on the cells.

To enlighten the discrepancies between the in vitro situation and the clinical situation, a more clinical approach using uncentrifuged and unfiltered toothpaste slurry was also performed. However, the findings of these additional experiments are not rational enough due to multiple weaknesses. Indeed, the cells reacted generally very sensitive to the toothpaste slurry up to a concentration of 12 %, regardless of the toothpaste used. Merely, when lower concentrations were used, differences were noticeable, supporting the findings when toothpaste-conditioned medium was used to stimulate the cells. One explanation might be that the toothpaste slurry, independent of the ingredients, directly damages the cells due to its foam and its thicker consistency. Additionally, it should be added that the toothpaste slurry was not sterile filtered and thus the cells were possibly exposed to microorganisms. Although this should not cause immediate cell damage due to the short contact time of 2 min. Overall, it can be said that the experimental approach using uncentrifuged and unfiltered toothpaste slurry might be clinically closer, however, too many weak points appear for valid statements.

The clinical implications may nevertheless be independent of the knowledge of which ingredients per se are responsible for the cytotoxic effects of some dentifrices. Some possible recommendations based on the results of this in vitro study are that children should use dentifrices that are designed for them and that dentifrices with the detergent cocamidopropyl betaine (without any other detergents) should be preferred amongst others. In summary, child dentifrices are more tolerable for the cells tested than has been documented for adult dentifrices. Approximately 12 % of dentifrices for children showed LC50 values below 1 % compared to 45 % of dentifrices for adults, when tested on oral fibroblasts [17]. Furthermore, LC50 values more than 90 % were shown in three child dentifrices, but a similar level has not been shown for fibroblasts when stimulated with adult dentifrices. Consequently, it appears to be essential to ensure that children do not use dentifrices from their parents [5] and rinsing the mouth with a small amount of water after tooth brushing might be beneficial for children with known sensitive mucosa.
